# *Salmonella enterica* biofilm-mediated dispersal by nitric oxide donors in association with cellulose nanocrystal hydrogels

**DOI:** 10.1186/s13568-015-0114-7

**Published:** 2015-05-23

**Authors:** Massimiliano Marvasi, Ian A Durie, Eric S McLamore, Diana C Vanegas, Prachee Chaturvedi

**Affiliations:** Department of Natural Sciences, School of Science and Technology, Middlesex University, The Burroughs, London, NW4 4BT UK; Soil and Water Science Department, University of Florida, Gainesville, FL USA; Agricultural and Biological Engineering Department, University of Florida, Gainesville, FL USA; Food Engineering Department, Universidad del Valle, Cali, Colombia; Department of Mechanical Engineering, University of Colorado, Denver, CO USA

**Keywords:** *Salmonella enterica*, Biofilm dispersal, Nitric oxide donor, Microelectrodes, Cellulose nanocrystals hydrogel

## Abstract

Protected by extracellular polymers, microbes within biofilms are significantly more resistant to disinfectants. Current research has been instrumental in identifying nitric oxide donors and hydrogels as potential disinfectant additives. Nitric oxide (NO) donors are considered a very promising molecule as biofilm dispersal agents and hydrogels have recently attracted a lot of interest due to their biocompatible properties and ability to form stable thin films. When the NO donor MAHMA NONOate was dissolved in phosphate saline buffer, it was able to reduce the biomass of well-established biofilms up to 15% for at least 24 h of contact time. Encapsulation of MAHMA NONOate and molsidomine within a hydrogel composed of cellulose nanocrystals (CNC) has shown a synergistic effect in dispersing well-established biofilms: after 2 h of exposure, moderate but significant dispersion was measured. After 6 h of exposure, the number of cells transitioning from the biofilm to the planktonic state was up to 0.6 log higher when compared with non-treated biofilms. To further explore the transport processes of NO donors within hydrogels, we measured the nitric oxide flux from gels, at 25°C for a composite of 0.1 µM MAHMA NONOate–CNC. Nitric oxide diffuses up to 500 µm from the hydrogel surface, with flux decreasing according to Fick’s law. 60% of NO was released from the hydrogel composite during the first 23 min. These data suggest that the combined treatments with nitric oxide donor and hydrogels may allow for new sustainable cleaning strategies.

**Electronic supplementary material:**

The online version of this article (doi:10.1186/s13568-015-0114-7) contains supplementary material, which is available to authorized users.

## Introduction

Biofilms formed on washing systems in produce production facilities can be recalcitrant reservoirs of human pathogens, which are difficult to control and can potentially cause costly outbreaks (Srey et al. 2013). Pathogens in biofilms are resistant to common disinfectants and contribute to an increased risk in contamination of produce by potentially contaminated water (Zhang and Mah 2008; Corcoran et al. 2014). Several disinfectants, antibiotics and messenger molecules have been studied for their ability to dislodge existing biofilms (Barraud et al. 2006, 2009a, 2012). Of these, nitric oxide (NO) appears to be very promising, and currently nitric oxide donors are used clinically. Nitric oxide is effective as a biofilm dispersant, functioning as a messenger rather than a generic poison (Barraud et al. 2006, 2009a, 2012; Marvasi et al. 2014). Nitric oxide can be delivered to biofilms by using donor molecules (Wang et al. 2005), and the application of nitric oxide donors has the same effect as the direct addition of nitric oxide gas (Barraud et al. 2009b). In bacteria, nitric oxide acts as an active signaling molecule at very low concentrations and is able to disperse preformed biofilms at nano- or picomolar concentration, indicating that truly minute quantities of the chemical are sufficient for dislodging much of the biofilm (Barraud et al. 2006, 2009a, 2012; Marvasi et al. 2014). Nitric oxide could have a universal effect on dispersal of bacteria biofilm including both Gram-positive and Gram-negative bacteria (Xiong and Liu 2010). The mechanisms of biofilm dispersion are not clear, but it appears to function in the transition of sessile biofilm organisms to free-swimming bacteria. For example, microarray studies have revealed that genes involved in adherence are downregulated in *Pseudomonas aeruginosa* upon exposure to nitric oxide donors (Firoved et al. 2004).

Over 105 nitric oxide donors have been characterized, and 6 have been currently tested as biofilm dispersal. Among these, the main studied are the sodium nitroprusside, (Barraud et al. 2006; Marvasi et al. 2014; Charville et al. 2008; Barraud et al. 2009a), molsidomine, diethylamine NONOate diethylammonium, NONOate diethylammonium, MAHMA nonoate (Marvasi et al. 2014), NORS (sodium nitrite citric acid) (Regev-Shoshani et al. 2013) and NO-releasing silica nanoparticles (Hetrick et al. 2009). Among these, MAHMA NONOate is one of the best candidates in biofilm dispersion: it spontaneously dissociates in a pH/temperature dependent manner, in serum it has a half-life of 1 and 3 min at 37°C and 22–25°C respectively, at pH 7.4 to liberate 2 mol of NO per mole of parent compound (Wang et al. 2005; Keefer et al. 1996; Hrabie et al. 1993). Interesting, when MAHMA NONOate was used to disperse *Salmonella enterica* and *Escherichia coli* biofilms (24-h old), up to 50% of *Salmonella* 14028 and a cocktail of six *Salmonella* outbreak strains biofilms were dispersed when incubated for 6 and 24 h at 22°C (both p < 0.0001). About 40% of dispersion was measured also for the pathogenic *Escherichia coli* O157:H7 when exposed at room temperature for 6 and 24 h (Marvasi et al. 2014).

Nitric oxide donors can be associated with other disinfectants obtaining a synergistic effect in terms of biofilm dispersion. For example, a 20-fold increase in the efficiency was observed when nitric oxide was applied together with chlorine in removing multispecies biofilms (Barraud et al. 2009b). In addition, we recently showed that the nitric oxide donor MAHMA NONOate and molsidomine were also able to increase up to 15% *Salmonella* biofilm dispersion when associated with the industrial disinfectant SaniDate 12.0 (Marvasi et al. 2014).

We used this foundation to hypothesize that nitric oxide donors can also be applied to hydrogels in order to obtain a synergistic effect in terms of biofilm dispersal. Hydrogels have recently attracted a lot of interest due to high strength/stiffness, optical transparency, biocompatibility, biodegradability, and highly porous structural network (Klemm et al. 2011; Siro and Plackett 2011) and the ability to carry bactericidal molecules (Azizi et al. 2013; Drogat et al. 2011; Feese et al. 2011). Hydrogels are water-swollen, and cross-linked polymeric network produced by the simple reaction of one or more monomers (Ahmed 2013). The industrial applications of hydrogels are extremely heterogeneous, ranging from agriculture, drug delivery systems, food additives, biomedical applications such as implants, and separation of biomolecules (Thakur and Thakur 2014).

Among the hydrogels, cellulose nanocrystals (CNC) is one of the most promising polymer because it is an eco-friendly product, cheap and safe (George et al. 2011). CNC has high crystallinity, high water holding capacity and excellent mechanical and thermal properties (George et al. 2011). For our scope, CNC was procured from a process development center that focuses on production of wood-derived renewable nanomaterials. CNC was selected as the hydrogel base because this material has an ionic charged surface appropriate for binding biofilm outer extracellular polymeric substances (EPS) (Marvasi et al. 2014; George et al. 2011; Marvasi et al. 2010).

The main objective of this study was to measure the synergistic effect of the nitric oxide donor MAHMA NANOate in association with CNC during nitric oxide-mediated biofilm dispersion of *S. enterica* sv Typhimurium. We focused our experiments on well-structured biofilms and we measured the shortest effective exposure. The nitric oxide release profile, diffusion and flux rate were also measured during MAHMA NONOate–CNC association.

## Materials and methods

### Bacterial strains and culture media

*Salmonellaenterica* serovar Typhimurium ATCC14028, was used in this study. pGFP-ON, a strongly fluorescent construct carrying GFP protein expressed from the Salmonella *dppA* promoter (Noel et al. 2010), was transformed into the strains of interest by electroporation.

All strains were maintained as frozen glycerol stocks, and were sub-cultured into Luria–Bertani medium with appropriate antibiotics (50 µg mL^−1^ kanamycin, 100 µg mL^−1^ ampicillin).

### Chemicals and materials for microelectrode preparation

Single layer graphene oxide (SLGO; 0.8 nm thickness, 1–5 µm diameter, 99% purity) was obtained from ACS Materials. Methanol, ascorbic acid and lead acetate were purchased from Fisher Scientific (Atlanta, GA, USA). Cerium (IV) oxide (nanoparticles dispersion, <25 nm particle size, 10 wt% in H_2_O) and chloroplatinic acid (8 wt%) were procured from Sigma-Aldrich (St. Louis, MO, USA). Nafion (5% aliphatic alcohol) and* o*-phenylenediamine were acquired from Acros organics (Newark, NJ, USA). Nitric oxide gas (CP grade 99%) was purchased from AirGas (Gainesville, FL, USA).

### Nitric oxide donors

MAHMA NONOate and molsidomine were purchased from Sigma-Aldrich (St. Luois, MO, USA). For each compound, 1 mmol L^−1^ stock solutions were prepared in phosphate-buffered saline (PBS), pH 7.3 (PBS, Fisher, Waltham, MA, USA) and aliquots were stored at −80°C. For the assays, serial dilutions were always prepared fresh in ice-cold PBS just before the experiments and used within 5 min of their preparation. The biofilm dispersion potential was tested on polystyrene 96-well plates (Fisher, Waltham, MA, USA).

### Biofilm formation and dispersal on plastics

Overnight Luria–Bertani cultures of *Salmonella S.* typhimurium 14028 or *S.* typhimurium 14028 pGFP-ON (with 100 µg mL^−1^ ampicillin) were diluted 1:100 in CFA medium as described previously (Teplitski et al. 2006), and 100 µL of the diluted cultures were aliquoted into wells of 96-well polypropylene and polystyrene plates (Fisher, Waltham, MA, USA). Plates with bacteria were incubated for 24 h or 1 week (well established biofilm) at 37°C inside a Ziploc bag to prevent dehydration.

### Measurement of biofilms dispersal

Upon completion of the incubation, the medium with planktonic bacteria was removed and serial dilutions of nitric oxide donors in PBS or CNC (in 200 µL) were added to the wells with biofilms. Dispersal experiments were conducted at 22°C for a time ranging from 1 to 24 h. The dispersion of biofilms treated with the nitric oxide donor dissolved in PBS was measured by staining the remaining biofilms with 1% crystal violet in ethanol, as described previously (O’Toole and Kolter 1998; Merritt et al. 2005).

Biofilms dispersion treated with CNC (or control, CNC + PBS) was measured by directly monitoring the increase of fluorescence of planktonic cells of *S.* typhimurium 14028 pGFP-ON. At the end of the period of exposure, 170 µL of CNC-nitric oxide solution were transferred into a black 96-wells plate and fluorescence was measured by using Victor-2 multimode plate reader with a 485 nm/535 nm excitation/emission filter (Perkin Elmer, Waltham, MA, USA). Increase of fluorescent intensity was used to represent an increase of detached cells.

### Luminescence tests

Effects of selected nitric oxide donors on light production by a constitutively luminescent *Salmonella* strain were characterized as indirect assessments of toxicity of the compounds. 1 mL of *S.* typhimurium 14028 pTIM2442 (harboring the *luxCDABE* driven by a strong constitutive phage, Alagely et al. 2011) were washed with BPS and mixed with opportune reagents (BPS, molsidomine, CNC) and grown in black polystyrene plates (Corning, New York, USA). Molsidomine was diluted in PBS (9.89 g L^−1^) or CNC (Fisher Scientific, Waltham, MA, USA) to final concentrations of 10 µmol L^−1^. PBS + CNC and PBS alone were used as a control. Luminescence of *S.* typhimurium 14028 pTIM2442 was measured over time using Victor-2 multimode plate reader (Perkin Elmer, Waltham, MA, USA). Each experiment included 12 replicas.

### CNC-nitric oxide preparation

1 mmol L^−1^ stock solutions of MAHMA NONOate or molsidomine were prepared in PBS, pH 7.3 (PBS, Fisher, Waltham, MA, USA). Final solutions to 1 µM were prepared by dispersing the nitric oxide stock solution in an opportune volume of CNC. The solution was further vortexed for 30 s and applied of prewashed biofilm formed into polypropylene plates.

### Nitric oxide measurement

NO microelectrodes were prepared using the methods in Chaturvedi et al. (2014). Pt/Ir microelectrodes (2 µm tip diameter, 5.1 cm length, 81 µm shaft diameter; MicroProbe, Inc., Gaithersburg, MD, USA) were rinsed in methanol prior to use. Nafion, nanoceria, nanoplatinum, and reduced graphene oxide were deposited based on the methods in Chaturvedi et al. (2014) and Vanegas et al. (2014). Briefly, microelectrodes were platinized via pulsed-sonoelectrodeposition at 10 mV for 60 cycles (each cycle consisted of 1 s electroplating pulse followed by 1 s sonication pulse) in a plating solution of 0.002% lead acetate and 1.44% chloroplatinic acid. Thereafter, electrodes were dip coated for 5 min in a suspension of 10 wt% cerium oxide with 0.8 wt% ascorbic acid and 0.2 wt% SLGO. Electrodes were then dried at 100°C for 20 min. Next, electrodes were dip coated in 5% Nafion and dried at 110°C for 20 min; this step was repeated twice. Finally, an OPD membrane was formed on the tip of the probe by polarizing the electrodes at 900 mV in a solution of 5 mM OPD and 0.1 mM ascorbic acid in PBS (pH 7.4) until a stable current was recorded (less than 2% variability) (Friedemann et al. 1996; Koehler et al. 2008; Porterfield et al. 2001). The working principle for the NO microelectrode is based on oxidation of NO at the surface of the nanocomposite, generating the nitrosonium cation (NO^+^) and one free electron based on Eq. 1 below; free electrons are detected as oxidative current versus a reference electrode

1 $$NO \, \to \, e^{ - } + \, NO^{ + } + \, OH^{ - } \to \, HONO \, \to \, NO_{2}^{ - } + \, H^{ + }$$

### NO microelectrode calibration and flux measurements

All measurements were performed at a working potential of +500 mV versus a reference electrode composed of a Ag/AgCl wire immersed in 3 M KCl and inserted into a half-cell microelectrode holder (World Precision Instruments, Inc., Sarasota, FL, USA). For electrode calibration, a PBS solution was polarized for 1 h at +900 mV; current output was measured at constant potential while successively injecting NO stock solution prepared based on Porterfield et al. (2001). Calibrated microsensors were used to measure NO flux in the self referencing (SR) modality as described in detail by McLamore and Porterfield (2011). The SR microsensor technique is designed to measure flux within the concentration boundary layer, and involves computer-controlled translation of a microelectrode between two positions separated by a known distance (dX). Differential concentration (dC) is recorded in real time and flux is then calculated using Fick’s first law of diffusion (J = −D × dC dX − 1). The diffusion coefficient (D) for NO used in all experiments was 2.21 × 10^−5^ cm^2^ s^−1^ based on and Zacharia and Deen (2005).

### Statistical analysis

The statistical package JMP (SAS) was used to infer the ANOVA analysis (p < 0.05). Tukey means separation analysis was inferred in order to group the means.

## Results

### Synergistic effect of the association of nitric oxide donors with CNC

The effect of MAHMA NONOate as biofilm dispersal was previously assessed in our recent publication in a range of biofilm-forming microorganisms of industrial and/or clinical significance including *S. enterica* (Marvasi et al. 2014). Our previous efforts were focused on studying young biofilms (maximum 24 h old) and exposure time up to 6 h. In order to focus on more applicative approaches of the nitric oxide donor technology, we tested the nitric oxide donors on well-structured biofilms (1-week old biofilms) and for a shorter contact time (up to 1 h of exposure).

**Figure 1:**
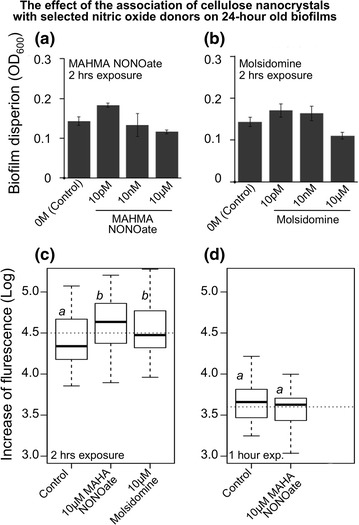
24-h old *Salmonella* biofilm dispersal by MAHMA NONOate dissolved in BPS and CNC. Biofilms were preformed by *S. enterica* typhimurium 14028 for 24 h on polystyrene prior to the treatment with MAHMA NONOate and molsidomine. Contact time at which biofilms were exposed to the nitric oxide donor is listed on each panel. **a**, **b** The decreasing of biofilm formation was measured by using the technique of crystal violet staining. **c**, **d** Fluorescent *Salmonella* 14028 pGFP-ON biofilm was treated with MAHMA NONOate and molsidomine dissolved in CNC. Increase in fluorescence of the planktonic cells was measured at 485 nm/535 nm. The *box-plots* encompass the lower and upper quartiles, *thick lines* within the *box* are the median values, and the *whiskers* indicate the degree of dispersion of the data. Outliers are shown as *dots*. *Dotted line* represents the grand mean. *Letters* above each *box* represent the Tukey means separation: *different letters* are significant different means.

As a first approach, we exposed a 24-h old *Salmonella* biofilm for a minimum of 2 h to different concentrations of MAHMA NONOate dissolved in PBS (10 µM, 10 nM and 10 pM): no significant results were measured (Figure 1a). The experiment was repeated with the nitric oxide donor, molsidomine, observing a similar result (Figure 1b). In further experiments, MAHMA NONOate or molsidomine were dissolved into CNC hydrogel to 10 µM (CNC–NONOate or CNC–molsidomine) to test whether the association of CNC was instrumental in increasing the dispersion potential of the nitric oxide donors within the 2 h. A fluorescent *Salmonella* 14028 pGFP-ON biofilm preformed on polypropylene was treated with 10 µM NONOate–CNC or 10 µM molsidomine–CNC. Fluorescence of the planktonic cells was measured after 2 and 1 h of exposure to the nitric oxide donors. The fraction of cells switching to the planktonic state was significant after 2 h of exposure (Figure 1c), but not significant after 1 h of exposure (Figure 1d).

To determine the viability of *Salmonella* during the exposure to CNC, the luminescence of *Salmonella* 14028 pTIM2442 upon association of CNC and molsidomine was measured. The general metabolic state of the cells was assessed by using the pTIM2442 system (Alagely et al. 2011). No variation in terms of viability was reported during a period of 8 h when CNC exposure was compared with the controls (Additional file 1: Figure S1).

**Figure 2:**
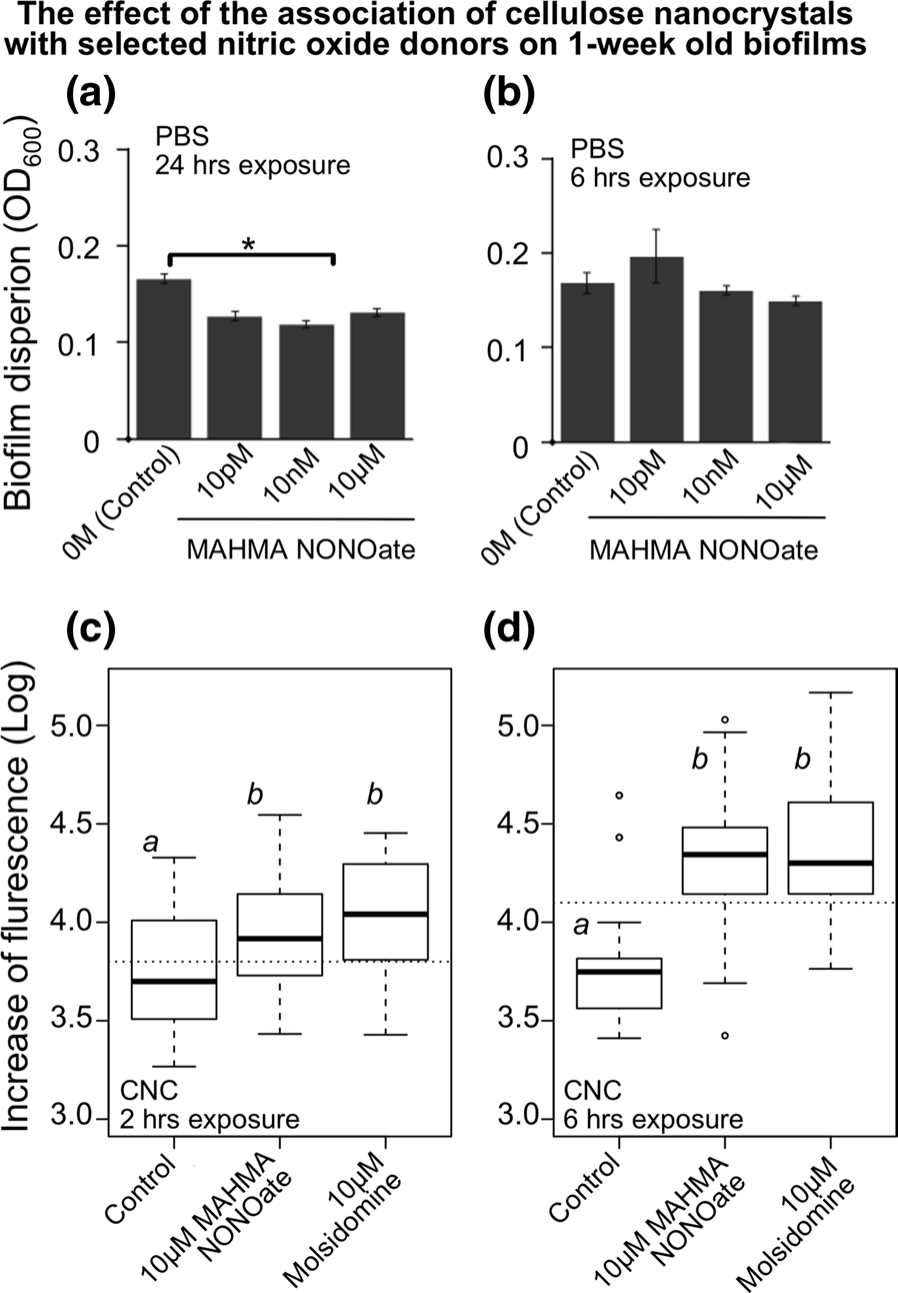
One-week old *Salmonella* biofilm dispersal by MAHMA NONOate dissolved in BPS and CNC. Biofilms were preformed by *S. enterica* typhimurium 14028 for 1-week on polystyrene prior to the treatment with MAHMA NONOate and molsidomine. Contact time at which biofilms were exposed to the nitric oxide donor is listed on each panel. **a**, **b** Decreasing of biofilm formation measured by using the technique of crystal violet staining. **c**, **d** A fluorescent *Salmonella* 14028 pGFP-ON biofilm was treated with MAHMA NONOate or molsidomine dissolved in CNC. Increase in fluorescence of the planktonic cells was measured at 485 nm/535 nm. The *box-plots* encompass the lower and upper quartiles, *thick lines* within the *box* are the median values, and the *whiskers* indicate the degree of dispersion of the data. Outliers are shown as *dots*. *Dotted line* represents the grand mean. *Letters* above each *box* represent the Tukey means separation: *different letters* are significant different means.

To measure the responsiveness of a well-established biofilm such as a 1-week old biofilm, *Salmonella* 14028 biofilms were exposed to different concentrations of MAHMA NONate dissolved in PBS for 24 and 6 h. 24 h of exposure to MAHMA NONOate reduced the biomass up to 15% (Figure 2a). However, 6 h of exposure did not significantly reduce the biomass (Figure 2b). As before, we tested whether the association of CNC was instrumental in increasing the dispersal potential of the nitric oxide donor. MAHMA NONOate and molsidomine were dissolved into CNC at a concentration of 10 µmol L^−1^. Interestingly, total fluorescence of the planktonic cells increased significantly in the wells with biofilms treated with both the donors (Figure 2c, d), reflecting an increase in cellular detachment at the tested concentrations. The biofilm exposed for 2 h showed an average of 0.3 log increase in fluorescence when compared with the control (PBS) (Figure 2c). When the same treatment was extended for 6 h, a difference up to 0.6 log was measured (Figure 2d). One-hour exposure was also tested but it was not significant (data not shown).

CNC was also tested on 1-week old biofilms at 4 and 37°C, to determine the effect of temperature on the dispersion potential. No significant differences were determined on 1-week old biofilm when exposed to molsidomine + CNC versus the control CNC + PBS. Biofilms were exposed for 6 h. 22°C seems to be the most appropriate temperature for obtaining a significant dispersal.

### Releasing profile of MAHMA NONOate in BPS and CNC

**Figure 3:**
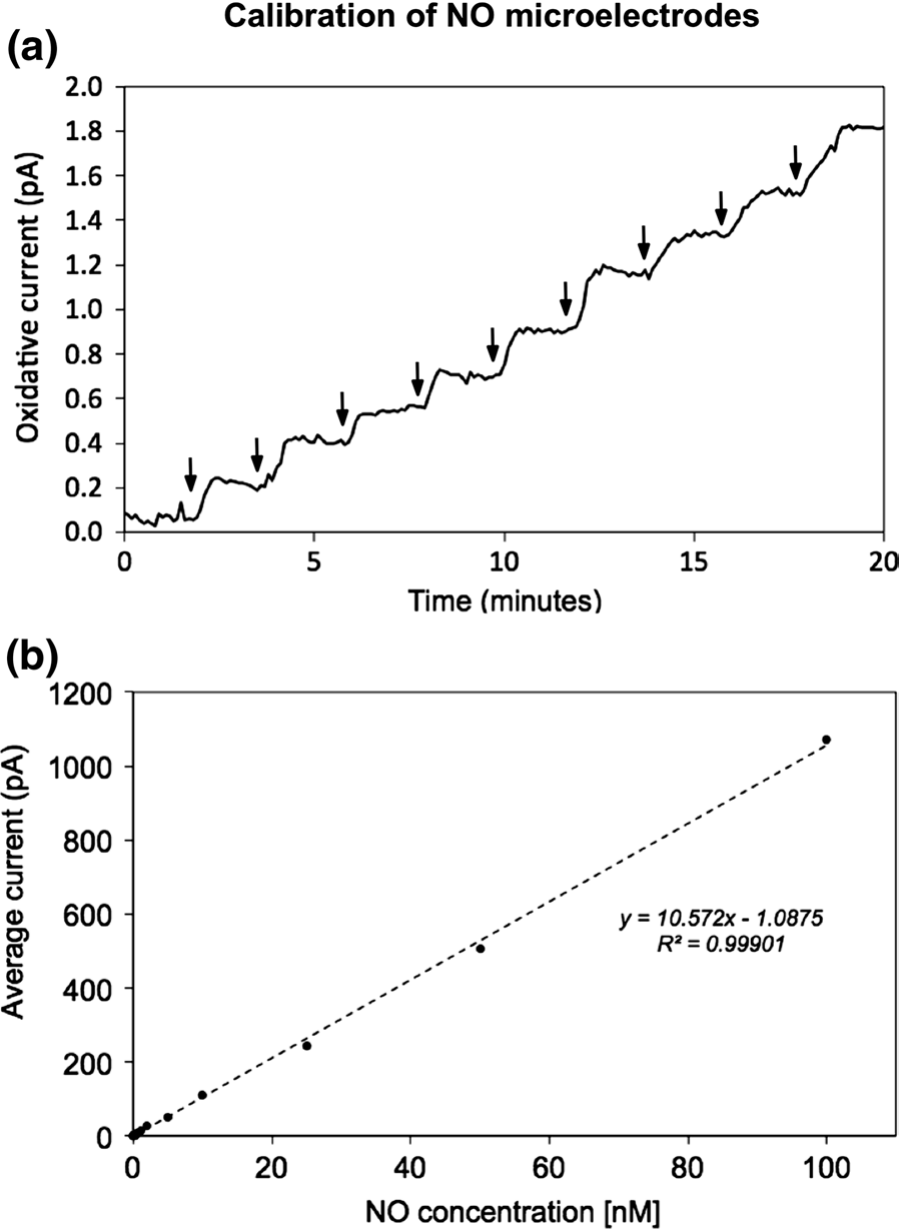
Calibration of NO microelectrodes. **a** Representative real time plot of NO microelectrode. *Vertical arrows* represent injection of NO stock solution. **b** Average output from three replicate microelectrodes (average sensitivity was 10.5 ± 0.1 pA nM^−1^).

To better understand the synergistic effect of CNC–MAHMA NONOate composites, the diffusion of NO from the hydrogel was studied in the absence of biofilms. Electrodes were first calibrated in buffer solution (Figure 3a, b). The average sensitivity of the NO microelectrodes was 10.5 ± 0.1 pA nM^−1^ within the range of 20 pM–100 nM. The average response time of the electrodes was 0.65 ± 0.24 s.

**Figure 4:**
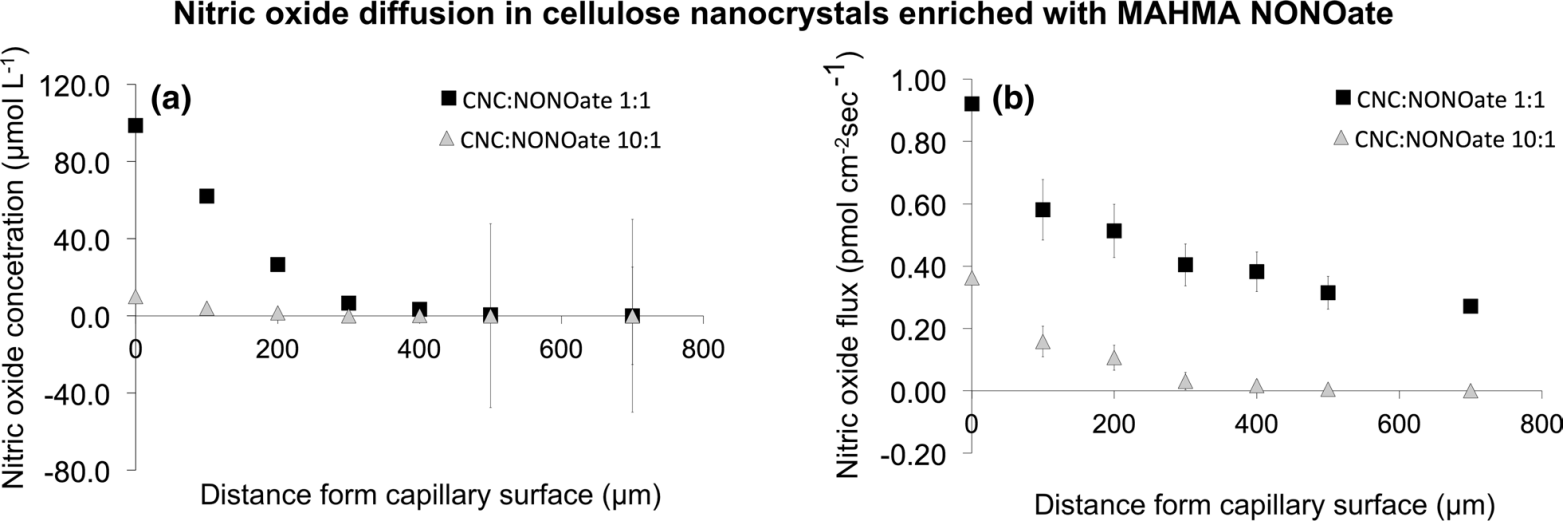
Nitric oxide diffusion in CNC. MAHMA NONOate was mixed with cellulose nanocrystals (*whisker type*) and a hydrogel was formed with a final concentration of 1 mM. A microcapilary was tapered with a tip diameter of ca. 10 μm using a glass puller. Consequently the hydrogel–NONOate was injected into the microcapillary and the microcapillary was placed into a phosphate buffer solution (PBS). A nitric oxide microsensor was immediately used to measure release of NO (both concentration and flux). **a** Nitric oxide concentration profiles from the hydrogel surface. **b** Nitric oxide flux profiles. 10:1 represent a 10 times dilution of the MAHMA NONOate dissolved in CNC. *Error bars* represent the standard error of three replicas.

Microelectrodes were used to directly measure NO flux from CNC hydrogels to better understand the mass transfer under abiotic conditions. A microelectrode was positioned at the surface of a hydrogel immediately after mixing the CNC and MAHMA NONOate while continuously measuring surface concentration and flux. At 25°C, nitric oxide dissociated from the MAHMA NONOate–CNC hydrogel. The surface concentration was 98.8 ± 0.1 µmol L^−1^ and the boundary layer was approximately 400 µm from the surface of the gel (Figure 4). The surface flux of nitric oxide was 0.92 ± 0.01 pmol cm^2^ s^−1^, and the decay profile was modeled using Fick’s first law (R = 0.989; Figure 4b) based on the methods in McLamore et al. (2009). As expected, the nitric oxide surface concentration (10.1 ± 0.6 µmol L^−1^), surface flux (0.36 ± 0.26 pmol cm^2^ s^−1^), and boundary layer thickness (approximately 150 µm) were significantly lower when the MAHMA NONOate was diluted 1:10 in the CNC hydrogel (Figure 4b). For the diluted sample, the regression coefficient correlating the measured flux profile and the predicted behavior using Fick’s first law decreased significantly (R = 0.713; Figure 4b).

**Figure 5:**
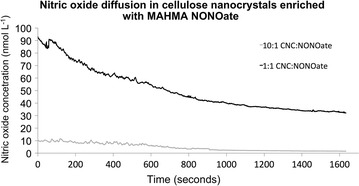
Nitric oxide release profiles of hydrogel-NONOate. Time 0 represents injection of the CNC:NONOate hydrogel into a glass capillary. 10:1 represent a 10 times dilution of the MAHMA NONOate dissolved in CNC. Experiment has been replicated three times. The *graph* represents an average of the three experiments.

Although the exact amount of nitric oxide liberated within biofilms from nitric oxide donors have not yet been established, we measured the release rate of nitric oxide from the CNC hydrogel enriched with MAHMA NONOate. MAHMA NONOate was initially dissolved in PBS to a concentration of 500 mM, and then it was dissolved in CNC at a final concentration of 1 µM. A nitric oxide microelectrode was used to measure the release rate (Figure 5). Our measurements (three replicas) showed that 60% of the nitric oxide is released as gas during the first 23 min (~725 s); the decay constant was 4.1 ± 0.4 h^−1^. As expected, a 1:10 dilution of MAHMA NONOate in CNC significantly decreased the surface concentration/flux, and the time required for approximately 60% of nitric oxide released as gas (~1125 s) was significantly longer; the decay constant was 2.3 ± 1.1 h^−1^.

## Discussion

Every wet surface is a substrate for microbial biofilms and in food handling facilities biofilms can be particularly problematic. Protected by extracellular polymers, microbes within biofilms are significantly more resistant to chlorine and other disinfectants (Corcoran et al. 2014). As a result, several disinfectants have been used to remove biofilm, however disinfectants fail to completely eradicate biofilms from food contact surface materials (Corcoran et al. 2014). Current research has been instrumental in identify nitric oxide as potential additive to disinfectant (Barraud et al. 2006; Marvasi et al. 2014).

To focus on applicative approaches of the nitric oxide donor technology, we focused on dispersing well-established biofilms in the shortest time possible. To meet this need, a composite of nitric oxide and a hydrogel was used, considering the attractive physicochemical properties of hydrgoels as reviewed by Malmsten (2011). Nitric oxide donors have been combined with a number of hydrogels in preliminary studies (Koehler et al. 2008), but to date no hydrogels have been developed with nitric oxide donors which can potentially be scaled up for commercial applications. In this work, CNC was selected as a candidate for potential large scale application because the material is produced in large batches by a commercial forest based nanomaterial processing facility. In addition, CNC has an ionic charged surface that can facilitate attachment of biofilm EPS (Marvasi et al. 2010). For the first time, we show that association of MAHMA NONOate and molsidomine with CNC improved the biofilm dispersion potential when compared with PBS (Figures 1, 2). CNC–MAHMA NONOate allowed the dispersion of 1-week old biofilm with a contact time of at least 2 h; This can be ascribed to the combination of two different factors: (1) the cells do not have enough time to switch from the biofilm state to the planktonic state in less than 2 h; (2) the nitric oxide diffusion into the biofilm requires a certain amount of time (based on concentration of the nitric oxide donor in the gel). Temperature seems also to play a role on the dispersion potential. In terms of dispersal the exposure to 22°C results to be the most appropriate temperature when compared with 4 and 37°C. We can hypothesize that at 37°C the nitric oxide is completely depleted within a short time. On the opposite at lower temperature the nitric oxide releasing prolife is reduced in CNC. It is well known that properties of CNC change according with the temperature (George et al. 2011).

Due to the porous network, diffusion is the predominant transport process within hydrogels and cell aggregates. We measured the nitric oxide diffusion from CNC, observing that nitric oxide can diffuse up to 500 µm from the capillary opening, which is an important observation since 1-week old biofilms rarely have a thickness greater than 0.5 mm (Paramonova et al. 2007). With reference of the releasing time, our measurements show that 60% of the nitric oxide is released as gas during the first 12 min. In the literature, similar release profiles in PBS were observed with the nitrosothiols* S*-nitroso-*N*-acetylpenicillamine (SNAP),* S*-nitroso-l-glutathione (GSNO) and sodium nitroprusside (Barraud et al. 2009b).

Finally, it is worth mentioning that the improved ability of MAHMA NONOate-CNC association to disperse biofilm may be due to the low-moderate antimicrobial activity of CNC (Azizi et al. 2013), even though in our experimental conditions, CNC did not show any significant antimicrobial activity. In literature other molecules have been associated with CNC to improve its antimicrobial effectiveness: for example, CNC stabilized with ZnO–Ag exhibited greater bactericidal activity against *Salmonella choleraesuis* and *Staphylococcus aureus* compared to cellulose-free ZnO–Ag heterostructure nanoparticles of the same particle size (Azizi et al. 2013). Association of porphyrin (Feese et al. 2011) and silver nanoparticles (Drogat et al. 2011).

Further studies should also be addressed in associating nitric oxide donors with nanoparticle composites with effective antibacterial and biofilm-disrupting properties. To that end several polymers can be used, for example silk fibroin–silver nanoparticle composite showed both an effective antibacterial activity against the methicillin-resistant *S. aureus* and as inhibitor of biofilm formation (Fei et al. 2013).

The mechanical and physical properties of the bio-composites can also be interesting to study in association with nitric oxide donors. It is well known that bacterial adhesion was sensitive to surface roughness and enhanced as the roughness of composite in catheters surfaces (Cheng et al. 2013). For example *Staphylococcus epidermidis* adhesion and growth were markedly higher on rough titanium surfaces than on smooth surfaces (Cheng et al. 2013). Nitric oxide may help in fostering a better dispersion over these surfaces.

To our knowledge, this is the first study that shows the association of nitric oxide donors with CNC as a biofilm dispersant agent. Further studies can determine the association of other additives and further applications in foaming solution, in addition to testing different hydrogels. This, in turn, can implement new sustainable cleaning strategies by expanding the tool-kit of pro-active practices for “Good Agricultural Practices (GAPs), “Hazard Analysis and Critical Control Point” (HACCP) and Cleaning-in-place protocols (CIP).

## Additional files

Additional file 1: Figure S1. Luminescence of *Salmonella* 14028 pTIM2442 upon exposure to CNC and molsidomine. General metabolic state of the cells was assessed using the redox-coupled FMNH2/Luciferase produced by a *S.* Typhimurium ATCC14028 strain harboring high copy number plasmid in which the *luxCDABE* operon is under the phage λ promoter (pTIM2442). Concentrations of molsidomine was 10 µM. Combination of molecules to which cultures of *Salmonella* 14028 pTIM2442 were exposed are listed on the figure. Each graph represents the average of 12 replicas.
