# Supplementary figures and images for: Salmonella enterica biofilm-mediated dispersal by nitric oxide donors in association with cellulose nanocrystal hydrogels
Source: AMB Express. 2015 May 23;5:28. doi: 10.1186/s13568-015-0114-7 (PMC4441645; doi:10.1186/s13568-015-0114-7)

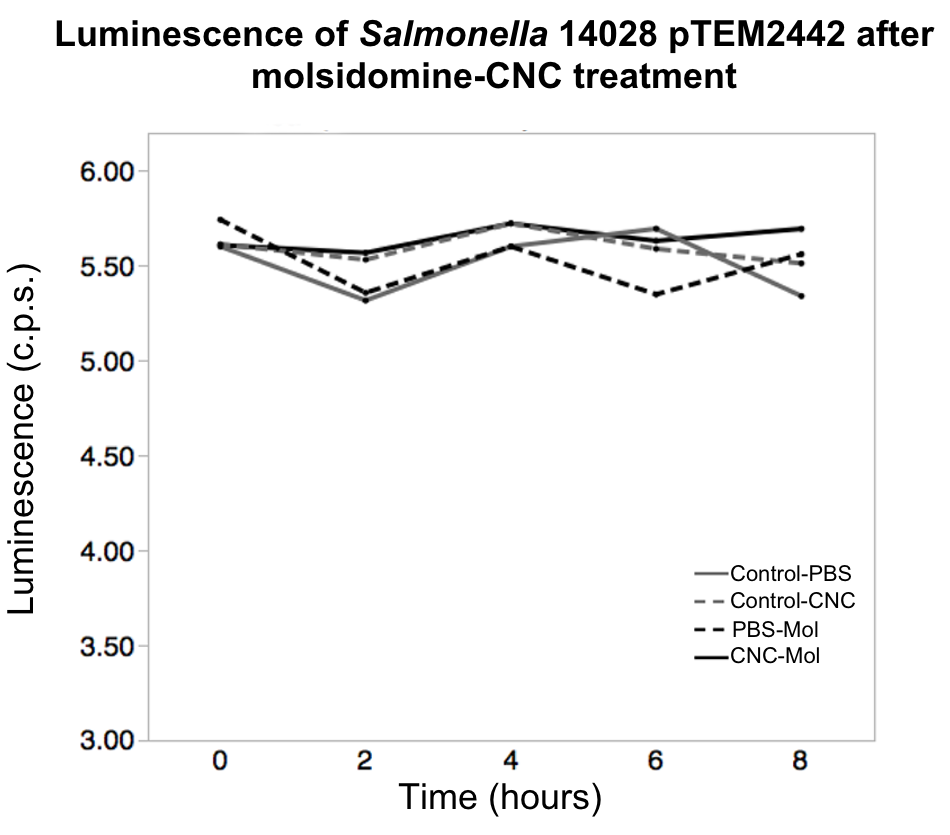

Supplement: Additional file 1: Figure S1. — Luminescence of Salmonella 14028 pTIM2442 upon exposure to CNC and molsidomine. General metabolic state of the cells was assessed using the redox-coupled FMNH2/Luciferase produced by a S. Typhimurium ATCC14028 strain harboring high copy number plasmid in which the luxCDABE operon is under the phage λ promoter (pTIM2442). Concentrations of molsidomine was 10 µM. Combination of molecules to which cultures of Salmonella 14028 pTIM2442 were exposed are listed on the figure. Each graph represents the average of 12 replicas. [file 13568_2015_114_MOESM1_ESM.tiff]
